# The kidney in cirrhosis with portal hypertension

**Published:** 2010-05-25

**Authors:** D Olteanu, D Lupu

**Affiliations:** Internal Medicine Ⅰ, University Emergency Hospital BucharestRomania

**Keywords:** portal hypertension, ascites, dilutional hyponatremia, hepatorenal syndrome

## Abstract

Ascites, dilutional hyponatremia and hepatorenal syndrome are three clinical manifestations of the same physiopathological disorder: cirrhotic portal hypertension, hyperproduction of nitric oxide, arterial vasodilation with reduction of efficient arterial volume, which have as consequences renal vasoconstriction, sympathetic stimulation, the stimulation of renin–angiotensin–aldosteron system and of vasopressin secretion. In dilutional hyponatremia, the selective receptor V2 (vasopressin 1) antagonists may be efficient according to Spanish and American specialists and also according to personal experience [6,9].

Abbreviations:Ly = lymphatic hyperproduction,Alb = hypoalbuminaemia, NO = nitric oxide,Sy = sympathetic (nervous) system,HRS = hepatorenal syndrome

In portal hypertension (PHT) there is a functional hemodynamic intrarenal disorder and a strong stimulation of renin–angiotensin–aldosterone system (RAAS) and also a high level of circulating vasopressin. PHT is the cause of local hyperproduction of nitric oxide (NO) and of other substances (adrenomedulin, glucagon) which are all strong arterial vasodilators.

This arterial vasodilation is dominant in splanchnic territory. The arterial vasodilation explains the reduction of the efficient arterial volume (EAV) and the tendency of the arterial pressure to drop. The reduced EAV strongly stimulates volume receptors with a strong sympathetic stimulation and also the stimulation of RAAS of vasopressin production and with renal cortical vasoconstriction [1–5,7].

These mechanisms have initially had an adaptative function in order to reestablish the EAV and the arterial pressure: the rise in plasma volume secondary to renal retention of H_2_O and Na+, systemic arterial vasoconstriction (except for splanchnic arteries) secondary to the stimulation of V1 vascular receptors by vasopressin, the rise in cardiac output [10].

In more advanced stages of cirrhosis, the same mechanisms, initially adaptative, become causes of pathology:

1. Ascites as a consequence of an excessive renal H_2_O and Na+ retention (–RAAS).
2. Dilutional hyponatraemia appears as a consequence of the V2 renal tubular receptors stimulation by vasopressin with renal selective retro resorption of water. Selective vasopressin 1(V1) receptor antagonists are efficient in many of these cases [6,7,9].
3. Hepatorenal syndrome which follows an extreme renal vasoconstriction.

In these later stages, EAV and arterial pressure are reduced and natriuria drops dramatically. The water and Na+ renal retention are predominantly distributed in the venous visceral territory (‘visceral sequestration’) and a normal EAV cannot be reestablished. Visceral capillary pressure raises which, in the presence of hypoalbuminaemia, results in peritoneal transudation of fluid and in hyperproduction of visceral lymph, resulting in ascites formation.

Renal hemodynamic disturbance begins before any ascites exists (Table 1). In this preascitic stage, renal sodium excretion is already impaired, but can be proved only when sodium intake is higher than normal. Renal vasoconstriction is not very strong and can be counterbalanced by the renal vasodilators mechanisms (prostaglandins and others). In the presence of ascites, renal vasoconstriction is more important and the Na+ renal excretion is impaired. In refractory ascites, mortality is higher and a hepatorenal syndrome (type Ⅱ) follows in 40% of the cases.

**Table 1:** Progressive alteration of creatinine and sodium excretion in portal hypertension [8]

Stage	Creatinine(serum)	Renal vasoconstrictor mechanisms(+)versus vasodilating mechanisms(–)	⬆ Na^+^ Intake	Normal Na^+^ intake	⬇ Na^+^ Intake	⬇ Na^+^ Intake and diuretics
*Preascitic*	N	+/––	+	N	N	N
*Moderate ascites*	N	+/–	+	+	+/–	N
*Important ascites*	N	++/–	+	+	+	N
*Refractory ascites*	1,2 –1,4 mg/dl, 40% HRS Ⅱ	**+++**/–	+	+	+	+
*Hepatorenal syndrome*	>1,5 mg/dl	+++	+	+	+	+

A refractory ascites appears in spontaneous bacterial peritonitis, dilutional hyponatremia and HRS type Ⅱ. In spontaneous bacterial peritonitis, aerobic Gram– negative bacteria are translocated from the intestine and can produce an inflammatory peritoneal response. They can also activate monocytes with the production of proinflamatory cytokines and supplementary NO, which increase arterial vasodilation. There is also an overexpression of Toll–like receptors and activation of NF–kB (nuclear factor kB) [10]. In these circumstances, the possibility of HRS occurrence increases. In dilutional hyponatraemia, V2 receptor antagonists obtained by cloning V2 receptors are efficient. A diuresis of 3–9 l/24h and 3–5 l after a single dose can be obtained with Satavaptan, urinary Na rises, ascites and edema are reduced [6,9]. These drugs also prevent the recurrences of ascites, diuretic induced hyponatraemia and may be useful in refractory cardiac insufficiency. V2 receptor antagonists prevent hepatic encephalopathy after hepatic transplantation.

When renal vasoconstriction is very strong and renal vasodilator mechanisms are overwhelmed by vasoconstrictor ones, hepatorenal syndrome (type Ⅰ, which presents as an acute renal failure, and type Ⅱ, which presents as a refractory ascites) [8]. When a HRS appears, some supplementary vasoconstrictors become active (Table 2): the rise in plasmatic level of endothelins (as an effect of endotoxinaemia) and a significant rise of the angiotensin Ⅱ, an intrarenal invasion of vasoconstrictors, as a consequence of a raised production of tromboxans, leukotriens and adenosine, and, also a reduced production of intrarenal vasodilators such as some prostaglandins and kallikrein (an imbalance in kinin–kallikrein equilibrium).

**Table 2:** Vasoactive intrarenal substances in HRS [5]

**Intrarenal vasodilators**	**Intrarenal vasoconstrictors**
Prostacyclin	Angiotensin Ⅱ
Prostaglandin E2	Norepinephrine
Nitric Oxid	Neuropeptide Z
Natriuretic atrial peptide	Endothelin Ⅰ
Kinin–kallikrein system	Adenosine
	A2 Thromboxane
	Cysteinyl Leukotriene
	F2 Isoprostane

The result is a formidable renal arterial vasoconstriction with an extreme ischemia, especially in the cortical area and with HRS.

HRS may also (rarely) develop in other liver diseases such as alcoholic hepatitis or in acute liver failure [5].

The three clinical syndromes (dilutional hyponatremia, ascites and HRS) are different manifestations of the same pathogenetic axis: PHT– NO–⬇ AEV ⬆RAAS and ⬆vasopressin, ⬆Sy (Figure 1). The three clinical syndromes are the manifestations of a continuous clinical spectrum, the extreme of which is the hepatorenal syndrome [5]. These clinical syndromes may present as a single syndrome (ascites), in association with two of them (ascites and HRS, ascites and hyponatraemia) or all three together (HRS, ascites, hyponatremia).

The therapeutic consequence of the above mentioned mechanisms is the necessity to maintain the EAV by i.v. infusion of albumin when= 9l of ascitic fluid is removed by paracenthesis and also i.v. albumin in spontaneous bacterial peritonitis, and the association of  i.v. albumin and vasoconstrictors in type Ⅰ HRS [3,5,8].

**Figure 1:**
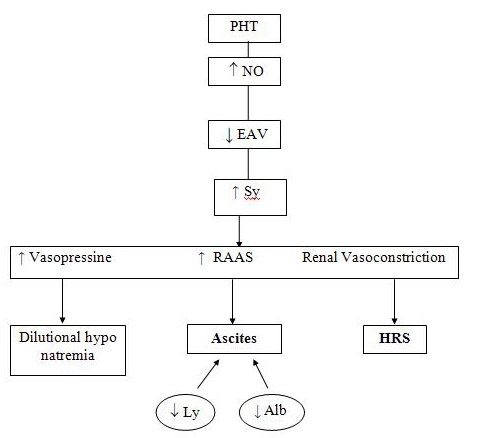
Clinical consequences of renal involvement in PHT
